# Zika virus and neurology: proving cause and effect

**DOI:** 10.1007/s00415-016-8165-5

**Published:** 2016-05-26

**Authors:** T. H. Massey, N. P. Robertson

**Affiliations:** Division of Psychological Medicine and Clinical Neuroscience, Department of Neurology, Cardiff University, University Hospital of Wales, Heath Park, Cardiff, CF14 4XN UK

## Introduction

‘Zika fever’ was first reported in Uganda and Tanzania in 1952. Caused by the Zika flavivirus it consisted of a mild and self-limiting illness of rash, low-grade fever, conjunctivitis, headache and myalgia. Two strains of Zika virus were subsequently identified (African and Asian) with transmission via mosquito or sexual contact. Since 2007 there have been several epidemics of the Asian strain including in French Polynesia (2013–2014; 20,000 cases) and Brazil (2015–2016; 30,000 cases), and these outbreaks have been associated with increased incidence of Guillain–Barré syndrome (GBS) in adults and microcephaly in infants.

Proving a causative link between an infectious agent and subsequent neurological damage is not straightforward. This month’s journal club examines three papers that attempt to provide evidence connecting Zika virus to neurological sequelae. The first is a case–control study of GBS during the Zika virus epidemic in French Polynesia. The second is a prospective cohort study reporting foetal growth defects in pregnant women in Brazil, some of whom had been infected with Zika virus. The third is a cellular paper looking at neural expression of AXL, a receptor that could mediate Zika virus entry into cells.

## Guillain–Barré syndrome outbreak associated with Zika virus infection in French Polynesia: a case–control study

GBS is a rare condition that encompasses various forms of acute immune-mediated polyradiculoneuropathy. It commonly presents as a monophasic, progressive symmetrical weakness with depressed or absent reflexes, often after an antecedent infection (*Campylobacter jejuni* gastroenteritis being the most common). French Polynesia (population of ~270,000 in 2013) typically reports between three and ten cases of GBS per year (i.e. 1–3 per 100,000), but during 4 months of the Zika epidemic (2013–2014) there were 42 cases (annualised incidence ~45 per 100,000). This paper compared these 42 consecutive GBS cases with two control groups from the same hospital: first, patients with non-febrile illness (*n* = 98); second, patients with confirmed Zika virus infection but no neurological sequelae (*n* = 70). Cases and controls were matched for age, sex, and island of residence.

GBS cases showed a male preponderance (74 %) with a median age of 42 years. Clinical characteristics included a viral prodrome (88 % of cases), lag of 4–10 days between viral syndrome and neurological symptoms, symmetric muscle weakness (79 %), areflexia or hyporeflexia (48 %), and facial palsy (79 %). Lumbar puncture showed increased protein only (93 %). Nerve conduction studies showed prolonged distal motor latencies (DMLs) and reduced compound muscle action potential (CMAP) amplitudes at diagnosis, with marked improvement at 3 months following treatment with intravenous immunoglobulin. Sensory potentials were unaffected. Clinical recovery was generally good (57 % walking unaided at 3 months), although 38 % required intensive care admission for ventilatory and/or nutritional support.

Virological testing revealed IgM against Zika (i.e. evidence of recent infection) in 93 % of GBS cases but just 17 % of non-febrile controls. However, there was no evidence of acute viraemia in any of the cases, consistent with patients rapidly clearing virus and being afebrile at presentation. Important negative serological tests included *C. jejuni*, HIV, CMV, and EBV. No association was found between previous dengue infection and GBS.

*Comment.* This paper shows a convincing correlation between the Zika epidemic in French Polynesia and increased incidence of GBS. The cases are consistent with the acute motor axonal neuropathy (AMAN) subtype of GBS, particularly the relatively preserved reflexes (52 %) and rapid recovery. Zika virus may well be the causative agent, but it is not possible to conclude this from a small observational study. Another unidentified (and untested) aetiological agent could exist. In addition, the controls were suboptimal: control group 1 was selected from non-febrile patients whereas 58 % of the GBS cases had fever as part of a viral prodrome; control group 2 (acute Zika infection) was not tested for Zika IgM/IgG nor followed up to assess for neurological sequelae.

Cao-Lormeau V-M et al (2016) Lancet 387:1531–1539.

## Zika virus infection in pregnant women in Rio de Janeiro—preliminary report

Phylogenetic analyses suggest that Zika virus was introduced to Brazil in late 2013. The first case was diagnosed in May 2015, and an increase in neonatal microcephaly identified in September 2015. To investigate a possible link between Zika infection and foetal abnormalities, this prospective cohort study recruited 88 pregnant women (gestational age 5–38 weeks) who presented with a new maculopapular rash during the epidemic of 2015. Seventy-two patients (82 %) were Zika-positive and had non-specific symptoms such as arthralgia, conjunctival injection, and pruritus. Standard pre-natal care in all 88 women documented immunity to rubella and CMV, a negative syphilis test, and positive dengue IgG (i.e. historical infection) in 88 %. Foetal ultrasonography was abnormal in 29 % Zika-positive women (*n* = 42), but normal in all Zika-negative women tested (*n* = 16). Various foetal abnormalities were observed including microcephaly (four cases), intrauterine growth restriction (IUGR), cerebral calcifications, oligohydramnios and cerebral blood flow abnormalities. Microcephaly, where present, tended to be part of a wider spectrum of neurodevelopmental problems. There was no specific correlation between gestational age at infection and foetal abnormalities. Interestingly, of the six babies born so far following abnormal ultrasound scans in utero, two have had microcephaly but in conjunction (and proportion) to IUGR, and another has done well following anhydramnios.

*Comment.* This study adds some weight to the hypothesis that Zika infection during pregnancy increases the risk of a ‘congenital Zika syndrome’ consisting of microcephaly and other neurodevelopmental problems. However, the study is incomplete (only 8 of 72 Zika-positive pregnancies have been completed), the numbers are small (particularly the control group), many Zika-positive women refused ultrasonography (42 %), and other causes of microcephaly have not been exhaustively investigated. In particular, other causes of rash (the study’s inclusion criterion) are not explored, and important possible confounding congenital infections such as acute dengue (measured by IgM) or HIV are not documented. In addition, many of the Zika-positive women were from rural, lower income areas, which could implicate other environmental factors.

Brasil P et al (2016) NEJM. doi:10.1056/NEJMoa1602412.

## Expression analysis highlights AXL as a candidate Zika virus entry receptor in neural stem cells

Zika virus can directly infect human cortical neural progenitor cells in vitro, cause transcriptional dysregulation, and attenuate cell growth. How Zika targets neural progenitors is unknown. The authors postulate that selective receptor expression in radial glia (neural stem cells of the developing brain) could mediate Zika virus entry, making these cells particularly susceptible to infection. Single-cell RNA sequencing of foetal human cerebral cortex (gestational week 39) enabled typing by genome-wide expression signatures, and assessment of putative Zika virus receptor expression. Multiple possible entry receptor genes were specifically enriched in radial glia, astrocytes, endothelial cells, and microglia. In particular, *AXL* (previously shown to mediate Zika and dengue virus entry into skin cells) was more highly expressed in radial glia than other receptors. Immunohistochemistry confirmed enrichment of AXL in radial glia and developing human retinal cells when compared to mature neurons. These expression patterns are conserved in mice and ferrets, and can also be seen in human iPSC-derived cerebral organoids suggesting model systems to assay inhibitors of Zika cell entry. A testable hypothesis that the neurotropism of the Zika virus is mediated via AXL is proposed.

*Comment.* This preliminary paper shows that AXL and numerous other putative Zika entry receptors are enriched in various neural cell types, particularly stem cells. It does not show any direct link between Zika virus and AXL, nor does it exclude any of the other receptors as mediators of Zika entry. Further work is needed to investigate how (and whether) AXL is involved in Zika neurotropism and stem cell selectivity, and whether blocking cell entry is a reasonable therapeutic avenue.

Nowakowski TJ et al (2016) Cell Stem Cell 18:1–6.

*Conclusion.* The timing of GBS a few days after Zika virus infection, and the discovery of foetal abnormalities following Zika infection in pregnancy similar to those seen in gestational rubella or CMV infections seem to incriminate Zika virus as the aetiological agent. It is, however, very difficult to prove a causative link, as these three papers illustrate. However, whilst larger studies are conducted a lack of scientific proof should not delay public health measures to reduce risk—for example, use of mosquito nets and insect repellent. It should also be remembered that even in the presence of Zika virus infection these remain rare neurological events: the approximate risks of GBS and microcephaly are 0.024 and 1 %, respectively.

